# Probing the enzymatic activity and maturation process of the EcAIII Ntn-amidohydrolase using local random mutagenesis

**DOI:** 10.3389/abp.2024.12299

**Published:** 2024-01-16

**Authors:** Joanna I. Loch, Anna Ściuk, Marta Kilichowska, Izabela Pieróg, Weronika Łukaszczyk, Katarzyna Zimowska, Mariusz Jaskolski

**Affiliations:** ^1^ Department of Crystal Chemistry and Crystal Physics, Faculty of Chemistry, Jagiellonian University, Krakow, Poland; ^2^ Doctoral School of Exact and Natural Sciences, Jagiellonian University, Krakow, Poland; ^3^ Institute of Bioorganic Chemistry, Polish Academy of Sciences, Poznan, Poland; ^4^ Department of Crystallography, Faculty of Chemistry, Adam Mickiewicz University, Poznan, Poland

**Keywords:** L-asparaginase, Ntn-hydrolase, random mutagenesis, enzyme engineering, AlphaFold prediction

## Abstract

This report describes a comprehensive approach to local random mutagenesis of the *E. coli* Ntn-amidohydrolase EcAIII, and supplements the results published earlier for the randomization series RDM1. Here, random mutagenesis was applied in the center of the EcAIII molecule, i.e., in the region important for substrate binding and its immediate neighborhood (series RDM2, RDM3, RDM7), in the vicinity of the catalytic threonine triplet (series RDM4, RDM5, RDM6), in the linker region (series RDM8), and in the sodium-binding (stabilization) loop (series RDM9). The results revealed that the majority of the new EcAIII variants have abolished or significantly reduced rate of autoprocessing, even if the mutation was not in a highly conserved sequence and structure regions. AlphaFold-predicted structures of the mutants suggest the role of selected residues in the positioning of the linker and stabilization of the scissile bond in precisely correct orientation, enabling the nucleophilic attack during the maturation process. The presented data highlight the details of EcAIII geometry that are important for the autoproteolytic maturation and for the catalytic mechanism in general, and can be treated as a guide for protein engineering experiments with other Ntn-hydrolases.

## Introduction

N-terminal-nucleophile amidohydrolases (Ntn-amidohydrolases) are enzymes that hydrolyze the amide bonds of amino acids, peptides, glycopeptides, and sphingolipids (Linhorst and Lübke, 2022), using an N-terminal residue as the primary nucleophile. Ntn-amidohydrolases are produced as inactive precursors which develop their enzymatic activity in an autocatalytic cleavage maturation process that releases the nucleophilic residue (Thr, Ser, Cys) at the newly formed N-terminus of subunit β (Michalska and Jaskolski, 2006). The *Escherichia coli* EcAIII protein is an Ntn-amidohydrolase with the L-asparaginase activity (Michalska et al., 2005). L-asparaginases are a large group of enzymes that convert L-Asn to L-Asp and ammonia. According to the most recent classification, EcAIII is a potassium-independent member of Class 2 L-asparaginases (Loch and Jaskolski, 2021; da Silva et al., 2022), formerly called plant-type L-asparaginases (Borek and Jaskólski, 2001).

**FIGURE 1:**
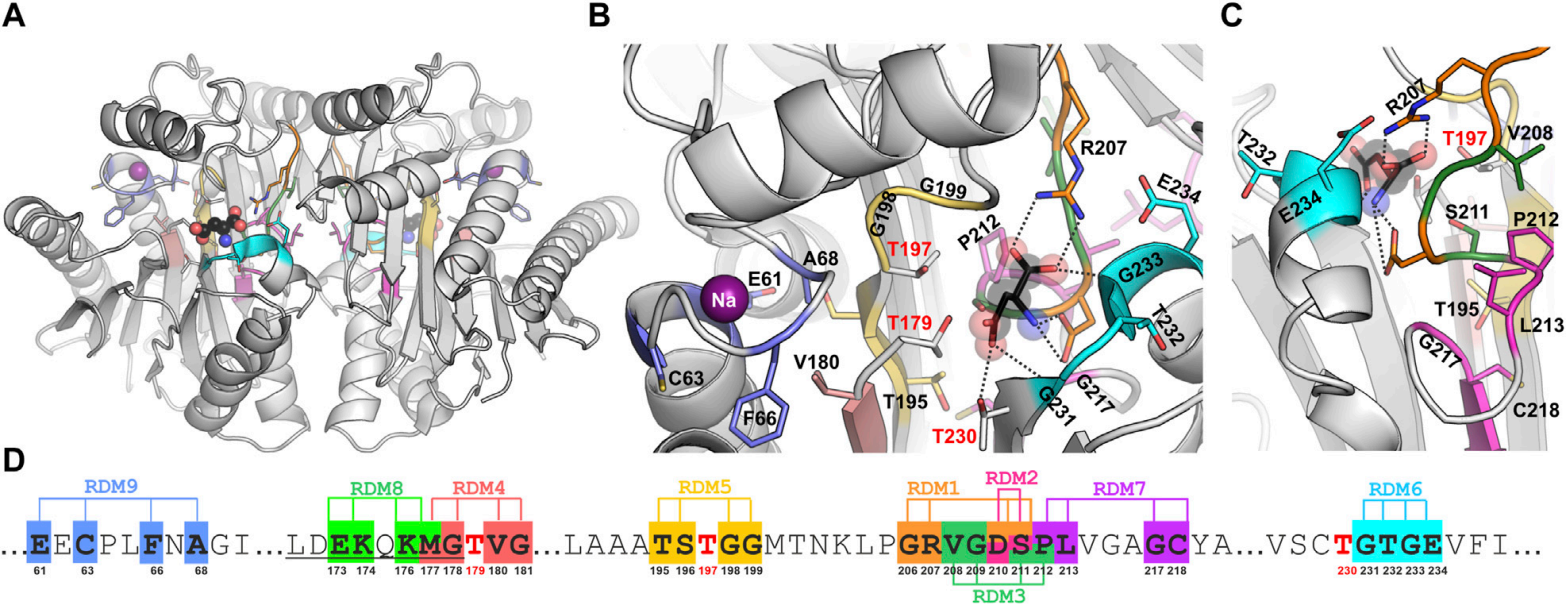
**(A)** Overall crystal structure of EcAIII (PDB: 2zal, gray) with product (L-Asp, ball-and-stick) bound in the active site; the violet spheres represent sodium ions coordinated in the stabilization loop. Panels **(B, C)** show detailed views (in different orientations) of the active site with color-coding of the randomization regions (the color code is the same as in Panel **(D)**); randomized residues are in stick representation. **(D)** A fragment of EcAIII sequence with randomization positions and numbers of the randomized residues marked below the sequence; sequence numbers of the Thr triplet are marked in red; the full EcAIII sequence is presented in Supplementary Figure S1.

The catalytic center of EcAIII is made of a threonine triplet: Thr179 (the nucleophile), Thr197, and Thr230. In the close vicinity of the triplet, other residues involved in substrate stabilization are located. Among them, the most important is Arg207, which acts as an anchor for the substrate α-carboxylate group, and Asp210, which stabilizes its amino group (Figure 1). The EcAIII molecule also features a sodium-binding (stabilization) loop, which is important for securing the proper orientation of Thr179 nucleophile (Michalska et al., 2008).

As all known Ntn-hydrolases, EcAIII requires a maturation step to develop its enzymatic activity. Upon maturation, the peptide bond between Gly178 and Thr179 is cleaved by the same Thr179 nucleophile (Michalska et al., 2008). The precursor protein is a homodimer, while the mature form of EcAIII is a dimer of two heterodimers, each comprised of subunit α and subunit β. After the autocleavage process, a flexible linker of ∼20 residues remains at the C-terminal part of subunit α. The linker is naturally disordered and undergoes degradation over time (Linhorst and Lübke, 2022).

Our recent studies of the impact of random substitutions (series RDM1, Supplementary Table S1) on EcAIII performance revealed that initiation of autocatalytic cleavage is a complex and multistep process, highly sensitive to even small changes in protein geometry and hydration pattern near the scissile Gly178-Thr179 bond (Loch et al., 2022). Following the previous run of local random mutagenesis, we performed further randomization trials to probe how the selected regions of the EcAIII molecule tolerate simultaneous multiple amino acid substitutions. Moreover, we also hoped to identify new EcAIII variant(s) with improved catalytic efficiency (k_cat_) or substrate affinity (K_m_), for potential utilization in leukemia therapy (Tosta Pérez et al., 2023).

In this work, segments of the EcAIII sequence for local random mutagenesis (Figure 1; Supplementary Table S1) were selected on the basis of crystal structure analysis. Residue randomization was carried out in the center of the EcAIII molecule, i.e., in the region important for substrate binding and its immediate neighborhood (series RDM2, RDM3, RDM7), in the vicinity of the catalytic threonine triplet (series RDM4, RDM5, RDM6), in the linker region (series RDM8) and in the sodium-binding loop (series RDM9). Although mutations usually led to inactive precursors, we were still able to identify several factors that affect the enzyme performance. Our report is one of few studies (Kim et al., 2010; Karamitros and Konrad, 2016; Xu et al., 2018; Loch et al., 2022) describing mutagenetic modification of Ntn-hydrolases.

## Methods

Random mutagenesis was performed according to the QuikChange protocol with the use of the mutagenic primers listed in Supplementary Table S1. In the naming scheme applied in this work, mutants are labeled as “RDMY-X,” where X is the ordinal number of a particular bacterial colony that was screened, and Y is the number of the mutagenesis trial (Supplementary Table S1; Figure 1). All SDS-PAGE gels marked with names of the mutants are presented at the end of Supplementary Material (as Supplementary Appendix – raw experimental data).

As a matrix for PCR reaction, the EcAIII sequence cloned to the pET11d (or pMCSG92) vector was used. After PCR reaction, the matrix was digested using *DpnI* enzyme (Thermo Scientific). Products of the PCR reaction were transformed to BL21 (DE3) Gold cells. Clone selection procedure, L-asparaginase activity test in cell lysate, and monitoring of autocleavage were performed according to previously described protocol (Loch et al., 2022). After monitoring of maturation by SDS-PAGE and testing for activity in cell lysate, several clones (active or inactive) were selected for plasmid DNA isolation and sequencing (Genomed SA, Poland) to check the mutations present.

Large-scale expression and purification of selected variants were performed according to previously described procedures (Loch et al., 2022, 2023). L-asparaginase activity of mutants was detected using Nessler method as describes earlier (Janicki et al., 2023). Structure prediction for selected mutants was made using AlphaFold2 (AF2) (Jumper et al., 2021) with the utilization of Amber relaxation refinement to assure the correct stereochemistry of the models.

## Results and discussion

The results presented herein are a continuation and complementation of the random mutagenesis report on EcAIII published previously as the RDM1 series (Loch et al., 2022). The randomization trials RDM2 - RDM9 were performed in eight selected regions of the EcAIII sequence (Figure 1; Supplementary Figures S1, S2). Screening was carried out manually for relatively small numbers of clones (Supplementary Table S2). The differences in the numbers of analyzed clones in each series were correlated with the analysis of the SDS-PAGE gels: if in a particular series a small number of clones were capable of autoprocessing, we did not continue the screening.

As the screening process was manual (not in a high-throughput mode), not all clones were submitted to sequencing due to a large number of plasmid isolations that would be too laborious for manual protocol. We decided to carry out sequencing for several clones from each series (Supplementary Table S3). The randomization trails allowed us to identify new EcAIII variants with interesting properties and to make several important observations related to the enzymatic activity of EcAIII, as well as to sequence variability observed in bacterial proteins annotated as Ntn-amidohydrolases (Janicki et al., 2023).

## Role of residues 210 and/or 211 (RDM2 series) in EcAIII activity

In the RDM1 mutagenesis trial, four residues at the substrate binding site, namely, Gly206, Arg207, Asp210, and Ser211, were randomized (Loch et al., 2022). In the RDM1 series 64 new EcAIII variants were analyzed. Most of them were not cleaved into subunits, and those which were processed did not exhibit L-asparaginase activity. In the RDM1 variants processed to subunits α and β (Loch et al., 2022), L-Asn hydrolysis was abolished by the absence of Arg207, which is crucial for substrate binding (Figure 1). In order to analyze the behavior of new variants with intact Arg207 (and Gly206), we decided to run the mutagenesis trial RDM2, in which only Asp210 and Ser211 were randomized (Supplementary Table S1).

Screening of 33 clones of the RDM2 series showed that only 11 variants were cleaved into subunits, and among them only eight retained the L-asparaginase activity (Supplementary Table S2). Sequencing revealed that six of the active clones had the wild type (WT) sequence of EcAIII, while the two other active clones carried the mutations D210A/S211P (RDM2-25) and D210P (RDM2-27) (Supplementary Table S3). We also found in the RDM2 series three clones that were processed to subunits but did not show L-asparaginase activity during screening. We sequenced two of them, and identified the following mutations: D210A (RDM2-17) and D210A/S211V (RDM2-32) (Supplementary Table S3).

**FIGURE 2:**
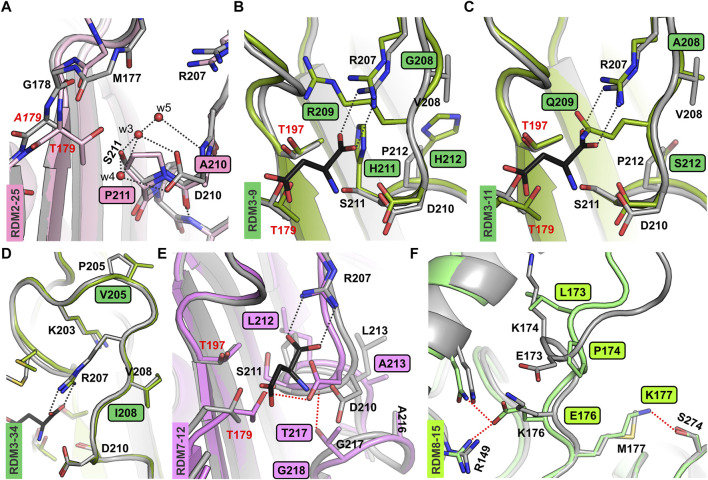
AF2-predicted structures of the new EcAIII variants. **(A)** Superposition of the RDM2-25 mutant (light pink) on the EcAIII precursor (PDB: 3c17, gray); water molecules (red spheres) present in the precursor structure are H-bonded (gray dashed lines) to Asp210 and Ser211; due to the mutations, this network of H-bonds is perturbed in the RDM2-25 mutant. **(B)** Superposition of the EcAIII structure (PDB: 2zal, gray) with bound L-Asp (black sticks) and the RDM3-9 mutant (dark green); the presence of Arg209 in the mutant affects the position of Arg207. **(C)** Structural changes in variant RDM3-11 (dark green); the side chain of Gln209 blocks the space reserved for the α-carboxylate group of the L-Asp substrate (black sticks, PDB:2zal). **(D)** Superposition of the RDM3-34 model (green) and WT EcAIII (PDB: 2zal; gray); there are no significant conformational changes. **(E)** Superposition of WT EcAIII (PDB: 2zal, gray) and the predicted model of mutant RDM7-12 (pink); the mutations resulted in atomic shifts and the formation of new H-bonds (red dotted lines), not present in the WT structure. **(F)** Superposition of the predicted structure of the EcAIII precursor (gray) and model of the RDM8-15 mutant (light green); major conformational changes were induced by the presence of Pro174 and Lys173; new H-bonds are marked by red dotted lines.

Although variants RDM2-25 and RDM2-27 were active, they had a very slow autoproteolysis rate. These observations indicate that the absence of Asp210 and/or Ser211 significantly slows down the maturation process. Although residues 210 and 211 do not seem to be directly involved in autoprocessing, as a polar residues they are part of an H-bond framework that assures the correct positioning of water molecules w3, w4, and w5 near the Gly178-Thr179 scissile bond, as visible in the crystal structure (Michalska et al., 2008) (Figure 2A). Although the AlphaFold2 models do not include water molecules, comparison of the predicted structures of mutants RDM2-25, RDM2-27, RDM2-17 and RDM2-32, and the crystal structure of the EcAIII precursor (PDB: 3c17) shows that in the absence of side chains Asp210 and/or Ser211 the correct network of water-mediated H-bonds near the nucleophilic Thr179 cannot be formed (Figure 2A). These observations can explain the slow autoproteolysis rate of the RDM2 mutants, as well as the low enzymatic performance, as Asp210 is also involved in substrate stabilization.

For the active mutants RDM2-25 and RDM2-27, large-scale expression and purification were carried out. We observed that only about ∼50% of the RDM2-25 and RDM2-27 mutant proteins were processed into subunits after 48 h. When autoprocessing progressed, we observed the development of L-asparaginase activity of variants RDM2-25 and RDM2-27. As it was impossible to separate the populations of mature and immature EcAIII molecules, we were not able to determine the kinetic parameters for preparations of partially cleaved proteins.

The data above suggest that mutations at positions 210 and 211 significantly reduce the autoprocessing rate, but mature variants develop L-asparaginase activity. On the other hand, reported earlier variants from the RDM1 series carrying similar mutations, e.g., RDM1-3, RDM1-12 or RDM1-29, did not exhibit any L-asparaginase activity at all (Loch et al., 2022). However, in the RDM1 mutants the Arg207 residue was absent, while the RDM2 mutants have intact Arg207 required as an anchor for the α-carboxylate group of the substrate. These observations indicate that the presence of Arg207 is absolutely necessary to maintain substrate affinity, while stabilization of the amino group of the substrate by a H-bond to Asp210 plays a less important role in substrate positioning.

## Random mutagenesis near the substrate binding site (RDM3 and RDM7)

For the RDM3 series, the SDS-PAGE analysis showed that most of the mutants were not cleaved into subunits. The only clones that were mature and active, RDM3-12 and RDM3-19, had sequence identical to WT EcAIII. We also made attempts to sequence several other immature RDM3 clones. Unfortunately, sequencing was successful only for three mutants (Supplementary Table S3): RDM3-9, RDM3-11, and RDM3-34. These three variants were produced at large scale and purified. Although at the screening stage we did not observe autoproteolysis, the large-scale purification revealed a very slow maturation process. Nevertheless, RDM3-9 and RDM3-11, remained enzymatically inactive, while variant RDM3-34 developed L-asparaginase activity. The predicted AF2 model of this variant did not reveal any significant conformational changes (Figure 2D), therefore, the reduced autoproteolytic efficiency of RDM3-34 has unknown origin.

Screening of the RDM3 mutants indicated that simultaneous mutations in the close neighborhood of Arg207 (positions 208, 209, 211, 212) is usually fatal for the catalytic activity of EcAIII because of conformational changes leading to incorrect positioning of the Arg207 side chain in the active site. In the RDM3 trial we randomized the highly conserved Gly209, while the rest of the mutated residues have only moderate or low conservation (Supplementary Figure S1). These observations indicate that Gly209 is important for maintaining the correct geometry of the active site. Gly209 is located very close to Arg207 and incorporation of residues with large side chains, such as Arg209 (in RDM3-9) or Gln209 (in RDM3-11) results in a positional shift of Arg207. An AF2 model of the RDM3-9 variant revealed that Arg209 and His211 strongly affect the position of Arg207, making substrate binding ineffective (Figure 2B). The predicted structure of mutant RDM3-11 reveled that Gln209 disturbs substrate binding by affecting its positioning (Figure 2C).

Most of the RDM7 clones, with randomization introduced at positions Pro212, Leu213, Gly217 and Cys218 (Figure 1), retained the ability to mature, but they lost their L-asparaginase activity. Most of active RDM7 clones (Supplementary Table S2) turned out after sequencing to be WT EcAIII. The predicted structure of inactive mutant RDM7-12 revealed a positional shift of the entire 207–217 loop, resulting in the formation of new H-bonds, the most important of which links Asp210 and Thr179 (Figure 2E). Such an interaction most probably disturbs substrate binding and affects the nucleophilic character of Thr179.

In the RDM7 series we detected a high proportion of WT sequences. A simple explanation might be an ineffective digestion of the PCR matrix by *DpnI*. However, we rather exclude this possibility as we always run control transformations in parallel. Another explanation might be that degenerated RDM7 primers with WT sequence were annealed in the PCR reaction to the DNA matrix with similar frequency as the degenerated oligonucleotides with non-complementary sequences. This observation suggest that the efficiency of random mutagenesis depends on the DNA sequence itself. In some (rare) cases, when it is not possible to produce new variants using only degenerated primers, other mutagenic approaches must be considered.

## Mutagenesis in conserved sequence regions (RDM4, RDM5, RDM6 and RDM9)

SDS-PAGE analysis of clones from the RDM4, RDM5, RDM6 and RDM9 series (Supplementary Table S1) revealed that in most cases the new EcAIII variants were incapable of splitting themselves into the α and β subunits. Absence of L-asparaginase activity was observed for all clones of RDM4 series. Plasmid sequencing revealed that selected clones in the RDM5 and RDM6 series that were active had the native WT EcAIII sequence (Supplementary Table S2). In series RDM4, RDM5 and RDM6, sequence randomization took place in the close vicinity of the conserved region of the threonine triplet (Thr179, Thr197, Thr230; Supplementary Figure S1). Mutants of the RDM9 series possessed random mutations in the conserved region of sodium-binding (stabilization) loop (Glu61, Cys63, Phe66, Ala68). In this series, the one active variant had sequence identical to WT protein (Supplementary Table S2).

These findings indicate that random mutagenesis in the highly conserved fragments of the sequence is ineffective, and it should be avoided or replaced by rational site-directed mutagenesis. However, our recent analysis of residue conservation near the catalytic triplet and the stabilization loop (Janicki et al., 2023) revealed that in bacterial EcAIII orthologs, substitutions in the Thr triplet and its close vicinity are possible but very rare. For example, the most variable position near Thr179 corresponds to Met177 in EcAIII sequence, which in other proteins can be substituted by Phe, His or Tyr, while the most mutation-prone position in the stabilization loop is Cys63 which can be replaced by Asp, Asn or Ser (Supplementary Figure S1).

## Mutagenesis within the variable linker region (RDM8)

It would seem that the chance of obtaining functional EcAIII variants via random local mutagenesis depends on the sequence element that is randomized: the more conserved the designated sequence fragment (associated with a particular function), the lower the probability of obtaining functional clones. From this point of view, the most promising segment is the least-conserved fragment of the EcAIII sequence, namely the flexible Glu159-Gly178 linker immediately preceding the nucleophilic Thr179 (Supplementary Figure S1). The linker does not participate in substrate binding and L-Asn hydrolysis of the mature enzyme. The role of the linker in autoproteolysis is not clear; however, recently we reported that correct positioning of the linker is essential for efficient autocleavage of the scissile Gly178-Thr179 bond (Loch et al., 2022).

The entire linker of ∼20 residues is usually not fully visible in the electron density maps, suggesting high mobility or progressive digestion. As the only available experimental model of EcAIII with part of the linker visible (residues 165–178) is the crystal structure of the T179A mutant (PDB: 3c17) (Michalska et al., 2008), we attempted to generate AF2 models of unprocessed EcAIII. The AF2 predicted conformation of the linker was significantly different from the 3c17 crystal structure. That structure of the inactive T179A mutant showed that residues Glu173 and Lys176 might be important for the stabilization of the scissile bond (which is a part of the linker), as Lys176 is H-bonded to Glu234 and Glu173 forms a salt bridge with Arg149 (Supplementary Figure S3). These interactions were absent in the predicted model of immature WT EcAIII. Instead, we observed an H-bond between the main chain carbonyl O atom of Glu173 and nitrogen of Lys176 (Supplementary Figure S3), as well as an H-bond between the side chains of these residues.

In the RDM8 series we randomized four residues: Glu173, Lys174, Lys176 and Met177, from the linker (Supplementary Table S1). Sequencing revealed that in the RDM8 trial four out of the seven active variants had WT sequence (Supplementary Table S2). However, clones RDM8-6 and RDM8-15 (and RDM8-17) carried mutations (Supplementary Table S3). SDS-PAGE analysis showed that in variants RDM8-6 and RDM8-15 autoprocessing was rather slow, however, ultimately the proteins were converted to mature αβ subunits and attained their L-asparaginase activity (as the active site region was not mutated). Variant RDM8-6 carried four mutations: E173I/K174Q/K176A/M177I. The AF2 model suggested that the K174Q substitution may lead to the creation of new H-bonds between Gln174, Gln152 and Gln19 (Supplementary Figure S3). According to the prediction, substitutions K174Q and E173I may result in a flip of the 171–173 side chains, affecting the Cα backbone trace of the linker.

For the RDM8-15 variant with the E173L/K174P/K176E/M177K mutations, the AF2 model suggested that significant conformational changes may be introduced in the linker by the presence of Glu176, H-bonded to Arg149 and Gln152, and by Lys177, H-bonded to Ser274 (Figure 2F), as these interactions are absent in the AF2 model of the WT protein. According to the AF2 model, the substitution K174P of RDM8-15 also contributes to the conformational changes of the linker. The conformational changes in the linker region analyzed in this section may potentially reduce autoproteolysis rate by hampering proper orientation of the Gly178-Thr179 scissile bond for nucleophilic attack.

The results of mutagenesis in the linker region show that by modifying this part of the EcAIII sequence one can tune the efficiency of autoproteolysis without “touching” the active site. This might be important for designing new EcAIII variants with controlled maturation process. Also, designing EcAIII mutants with impaired autoproteolytic activity might be useful for crystallographic modeling of the intact linker structure, as an important step towards the elucidation of the mechanism of autoproteolytic activation.

## Conclusion

This report describes a comprehensive approach to random mutagenesis of EcAIII and is a sequel to a previously published work (Loch et al., 2022). Our studies reveled that most of the new EcAIII variants had abolished or significantly reduced rate of autoprocessing, and were in consequence devoid of L-asparaginase activity. In this respect, we have demonstrated that local random mutagenesis of the EcAIII sequence is rather ineffective for designing better enzymes with increased substrate affinity. The problem lies not in the randomization itself, but is rooted in the biochemistry of Ntn-amidohydrolases. These enzymes are produced as inactive precursors and it is usually difficult to predict if the introduced mutations will affect the maturation process only, or L-Asn hydrolysis as well. Even if a given substitution could be beneficial for the ultimate substrate hydrolysis, this effect may not be observed if the same mutation is fatal to enzyme maturation. The only way to avoid such problems is to use circularly permutated genes for Ntn-amidohydrolase mutagenesis. This approach has been used only once, for the human HsAIII enzyme (Li et al., 2012).

We also predicted the role of several residues in the proper orientation of the linker and the scissile bond for autoprocessing. Moreover, we generated genes of several new active and inactive EcAIII variants that enrich the available library of EcAIII mutants (Michalska et al., 2008; Loch et al., 2022; Janicki et al., 2023). We believe that our randomization studies will help to better understand the catalytic properties of Class 2 L-asparaginases and will guide further engineering experiments with other Ntn-amidohydrolases as well as mechanistic studies (Andjelkovic et al., 2023) of the new Ntn-hydrolases.

## Data Availability

The original contributions presented in the study are included in the article/Supplementary Material, further inquiries can be directed to the corresponding author.
