# Supplementary material for: Probing the enzymatic activity and maturation process of the EcAIII Ntn-amidohydrolase using local random mutagenesis
Source: Acta Biochim Pol. 2024 Jan 16;71:12299. doi: 10.3389/abp.2024.12299 (PMC11077353; doi:10.3389/abp.2024.12299)

Supplementary tables

Table S1. Primer sequences used for mutagenesis.

series	primer sequences (FOR and REV, sequence: 5' → 3')	protein region
**RDM1***	CAATAAATTACCC**NNNNNN**GTTGGC**NNNNNN**CCCTTAGTGGGTG<br>CACCCACTAAGGG**NNNNNN**GCCAAC**NNNNNN**GGGTAATTTATTG	**Substrate binding site:**<br>Gly206, Arg207, Asp210 and Ser211
**RDM2**	CCGGACGAGTTGGC**NNNNNN**CCCTTAGTGGGTGCCGG<br>CCGGCACCCACTAAGGG**NNNNNN**GCCAACTCGTCCGG	**Substrate binding site:**<br>Asp210 and Ser211
**RDM3**	GACCAATAAATTACCCGGACGA**NNNNNN**GAT**NNNNNN**TTAGTGGG<br>CCCACTAAGGGACTATC**NNNNNN**TCG**NNNNNN**TAATTTATTGGTC	**Substrate binding site; Arg207 neighborhood (Arg207 is anchor for substrate):**<br>Leu208, Gly209, Ser211, Pro212
**RDM4**	GATGAAAAACAGAAA**NNNNNN**ACC**NNNNNN**GCCGTGGCGTTGG<br>CCAACGCCACGGC**NNNNNN**GGT**NNNNNN**TTTCTGTTTTTCATC	**Threonine triplet; Thr179 (nucleophile) neighborhood:**<br>Met177, Gly178, Val180, Gly181
**RDM5**	CAATTTGGCGGCAGCC**NNNNNN**ACA**NNNNNN**ATGACCAATAAATTAC<br>GTAATTTATTGGTCAT**NNNNNN**TGT**NNNNNN**GGCTGCCGCCAAATTG	**Threonine triplet; Thr197 neighborhood:**<br>Thr195, Ser196, Gly198, Gly199
**RDM6**	CGGTTTCTTGT**NNN**GGC**NNNNNNNNN**GTCTTCATCCGC<br>GCGGATGAAGAC**NNNNNNNNN**GCC**NNN**ACAAGAAACCG	**Threonine triplet (and oxyanion hole for substrate); Thr230 neighborhood:**<br>Gly231, Thr232, Gly233, Glu234
**RDM7**	GTTGGCGATAGT**NNNNNN**GTGGGT**NNNNNN**TGCTACGCCAAT<br>ATTGGCGTAGCA**NNNNNN**ACCCAC**NNNNNN**ACTATCGCCAAC	**Substrate binding site, loop near the active site:**<br>Pro212, Leu213, Gly217, Cys218
**RDM8**	CGCCACTGGAT**NNNNNN**CAG**NNNNNN**GGCACCGTGGGGG<br>CCCCCACGGTGCC**NNNNNN**CTG**NNNNNN**ATCCAGTGGCG	**Linker region:**<br>Glu173, Lys174, Lys176, Met177
**RDM9**	CGTCTGCTG**NNN**GAG**NNN**CCACTG**NNN**AAC**NNN**GGAATTGGC<br>GCCAATTCC**NNN**GTT**NNN**CAGTGG**NNN**CTC**NNN**CAGCAGACG	**Stabilization loop:**<br>Glu61, Cys63, Phe66, Ala68

*published previously (Loch *et al.*, 2022)

Table S2. Statistics of clone screening and sequencing.

RDM series	no. of analyzed clones	processed	active	STOP codon		RDM series	no. of sequenced clones	no. of clones with mutations	no. of clones with no mutations/WT	sequencing error
**RDM2**	**33**	11	8	2		**RDM2**	**10**	4	6	0
**RDM3**	**48**	4	3	3		**RDM3**	**8**	3	2	3
**RDM4**	**56**	3	0	7		**RDM4**	**0**	0	-	-
**RDM5**	**57**	6	1	8		**RDM5**	**1**	0	1	0
**RDM6**	**59**	4	2	8		**RDM6**	**1**	0	1	0
**RDM7**	**27**	23	11	0		**RDM7**	**7**	1	6	0
**RDM8**	**23**	7	7	6		**RDM8**	**7**	3	4	0
**RDM9**	**24**	2	1	0		**RDM9**	**1**	0	1	0

Table S3. Results of sequencing of selected clones. The naming scheme of mutants originates from the number of clone analyzed by SDS-PAGE (see: Appendix – Raw Data – at the end of Supplementary materials).

RDM series	results of screening: autoprocessing	screening: L-Asn hydrolysis	Detected mutations	Large scale purification, observations
**randomization trial RDM2 (vector pET11d)**				
RDM2-17	processed	not active	D210A	slow autoprocessing, no L-Asn hydrolysis
RDM2-25	processed	active	D210A, S211P	slow autoprocessing, L-Asn hydrolysis
RDM2-27	processed	active	D210P	slow autoprocessing, L-Asn hydrolysis
RDM2-32	not processed	not active	D210A, S211V	slow autoprocessing, no L-Asn hydrolysis
**randomization trial RDM3 (vector pMCSG92)**				
RDM3-9	not processed	not active	V208G, G209R, S211H, P212H	slow autoprocessing, no L-Asn hydrolysis
RDM3-11	not processed	not active	V208A, G209Q, P212S	-
**randomization trial RDM3 (vector pET11d)**				
RDM3-34	not processed	not active	P205V*, V208I	slow autoprocessing, L-Asn hydrolysis
**randomization trial RDM7 (vector pET11d)**				
RDM7-12	processed	not active	P212L, L213A, A217T, C218G	-
**randomization trial RDM8 (vector pET11d)**				
RDM8-6	processed	active	E173I, K174Q; K176A, M177I	-
RDM8-15	processed	active	E173L, K174P, K176E, M177K	-
RDM8-17	processed	active		-

*unexpected mutation

Supplementary figures

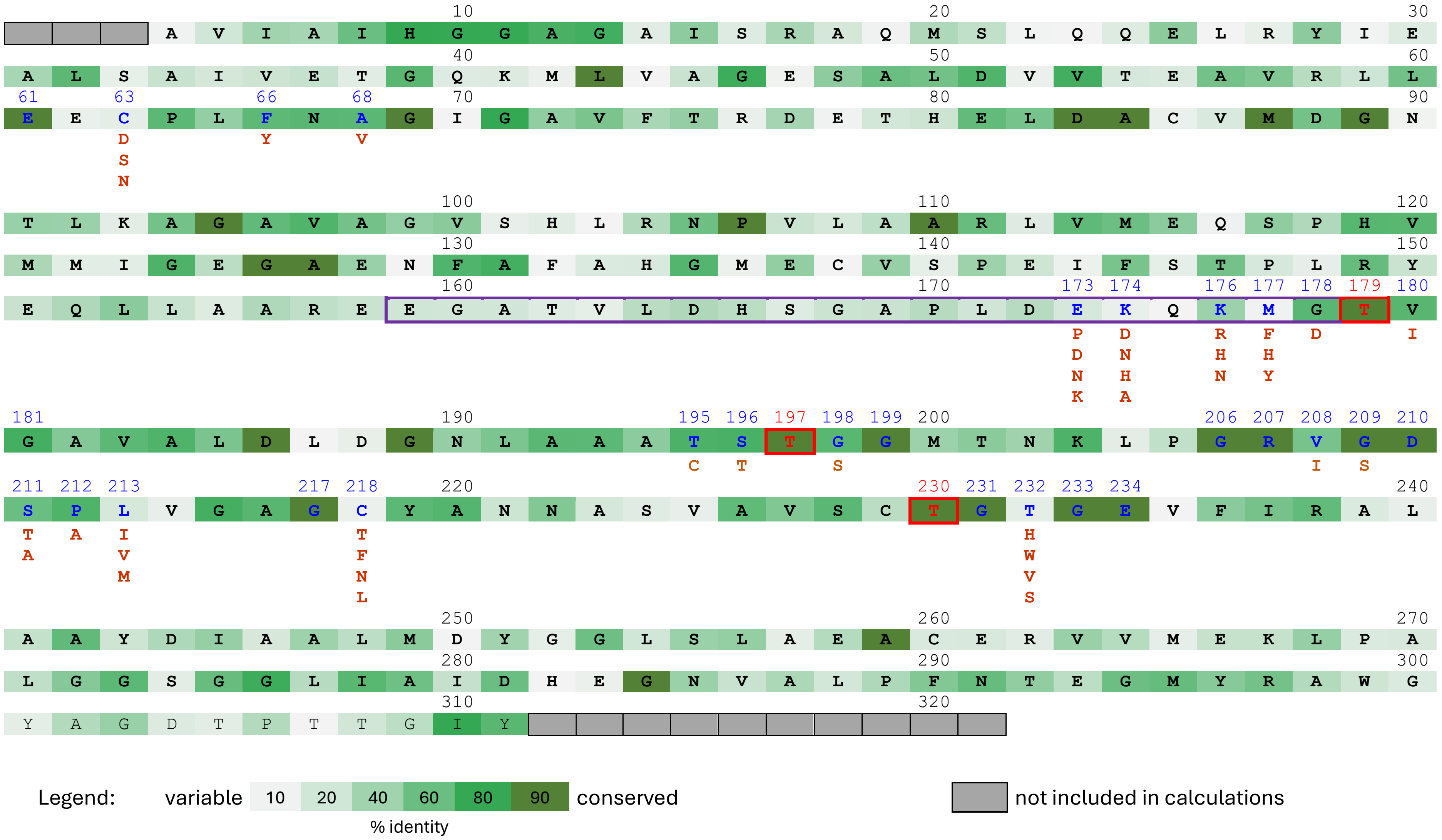

Fig. S1. Variable and conserved residues in the EcAIII sequence. Conservation of residues was defined on the basis of position-specific score matrix (PSSM) of Class 2 bacterial L-asparaginases calculated previously with EcAIII as the reference (Janicki *et al.*, 2023). Residue was marked as highly conserved (dark green) when percent of identity in other bacterial proteins was higher than 90%, while variable residues had less than 20% identity (light green). Calculations were performed for residues that are visible in the electron density map in the reference PDB structure PDB: 2zal, therefore several residues at the N- and C-termini were not included in calculations. Residues from the threonine triplet are marked in red (text, number, and outline); residues from the flexible linker are outlined in violet; residues used in random mutagenesis are marked in blue (text and number); brown amino acid symbols below selected residues show possible substitutions at that position found in other bacterial Class 2 L-asparaginases (Janicki *et al.*, 2023).

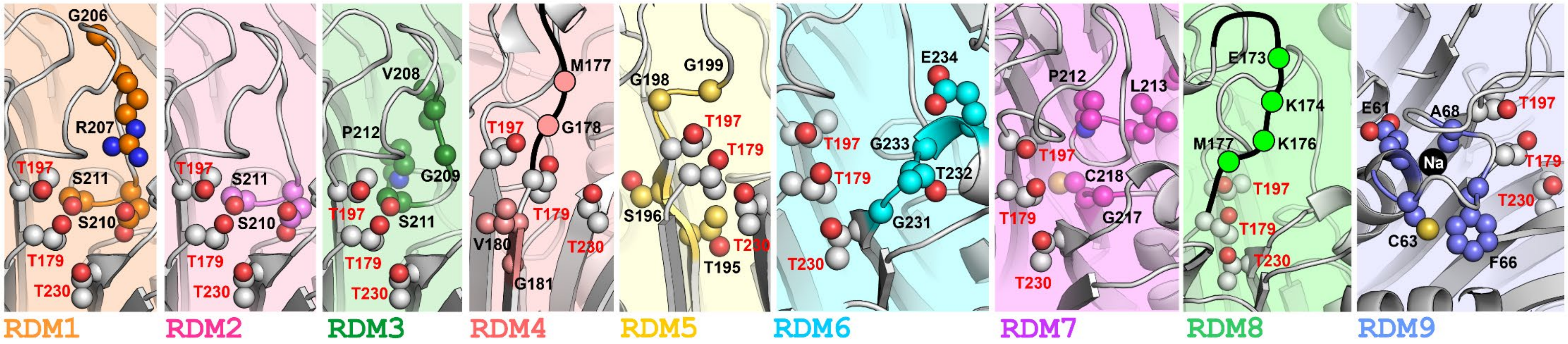

Fig. S2. Random mutagenesis sites RDM1 (Loch *et al.*, 2022) and RDM2-RDM9 shown separately. The linker region (not visible in the PDB structure 2zal) in RDM3 and RDM8 is marked by black solid line, while residues being part of linker are marked by solid circles. Residues from threonine triplet are marked in red.

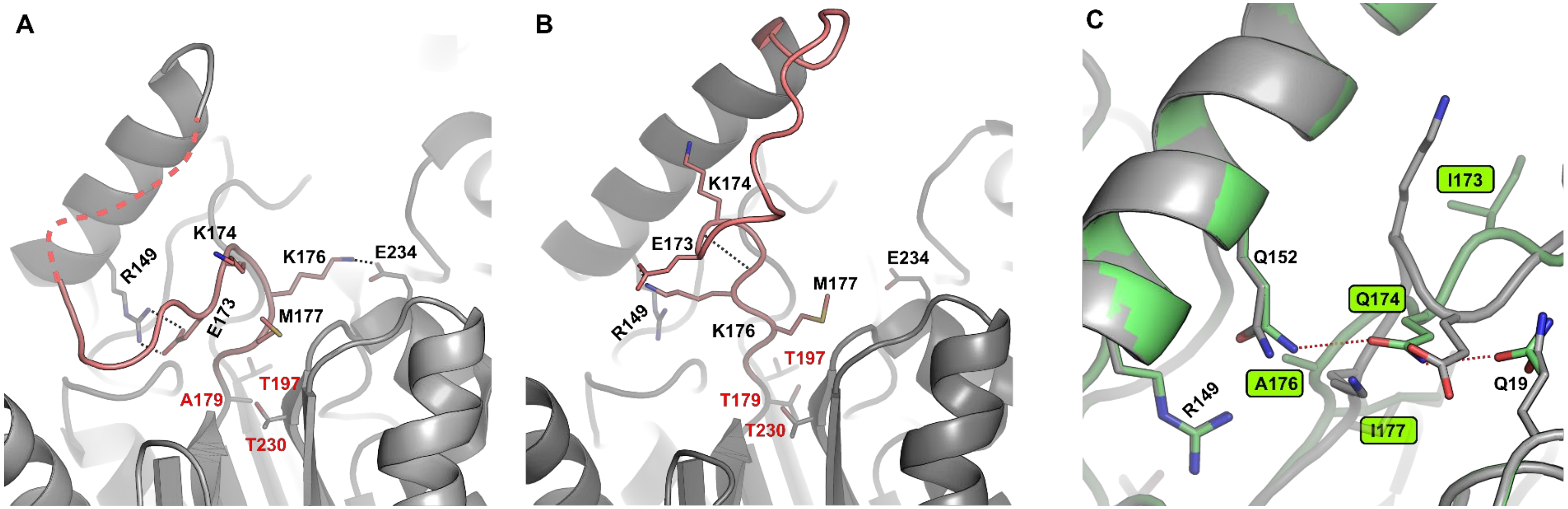

Fig. S3. (**A**) Position of the linker (light red) in the crystal structure of the EcAIII mutant T179A (PDB: 3c17); missing residues are marked by red dashed line. (**B**) Conformation of the linker (light red) in the WT EcAIII precursor model (grey) predicted by AF2; H-bonds are marked as black dotted lines. (C) Superposition of predicted AF2 model of variant RDM8-6 (green) and AF2 model of WT EcAIII (grey); new H-bonds between Gln174, Gln152 and Gln19 are marked by red dashed lines.

Supplementary references

Janicki, M., Ściuk, A., Zielezinski, A., Ruszkowski, M., Ludwików, A., Karlowski, W. M., Jaskolski, M. & Loch, J. I. (2023). *Protein Sci.* **32**, e4647.
Loch, J. I., Klonecka, A., Kądziołka, K., Bonarek, P., Barciszewski, J., Imiolczyk, B., Brzezinski, K., Gilski, M. & Jaskolski, M. (2022). *Acta Cryst.* **78**, 911–926.

Appendix – raw experimental data – SDS-PAGE gels from clone screening

1. SDS-PAGE gels from screening of RDM2 series.

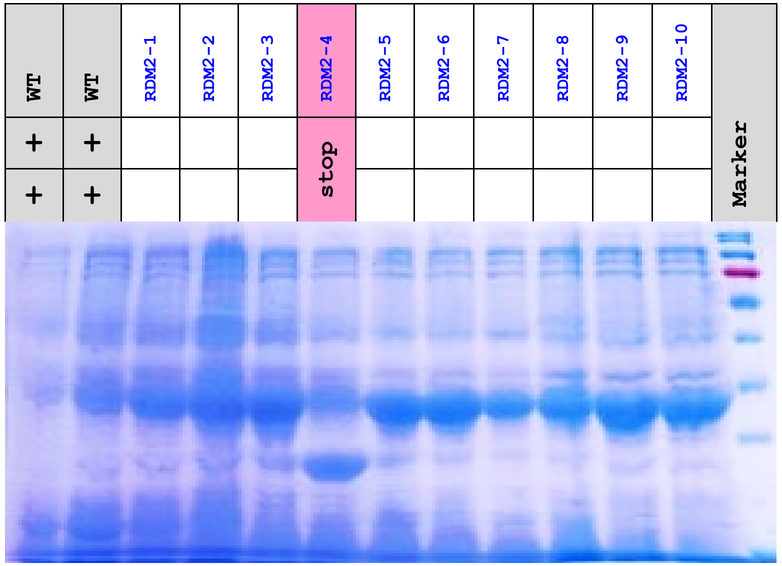

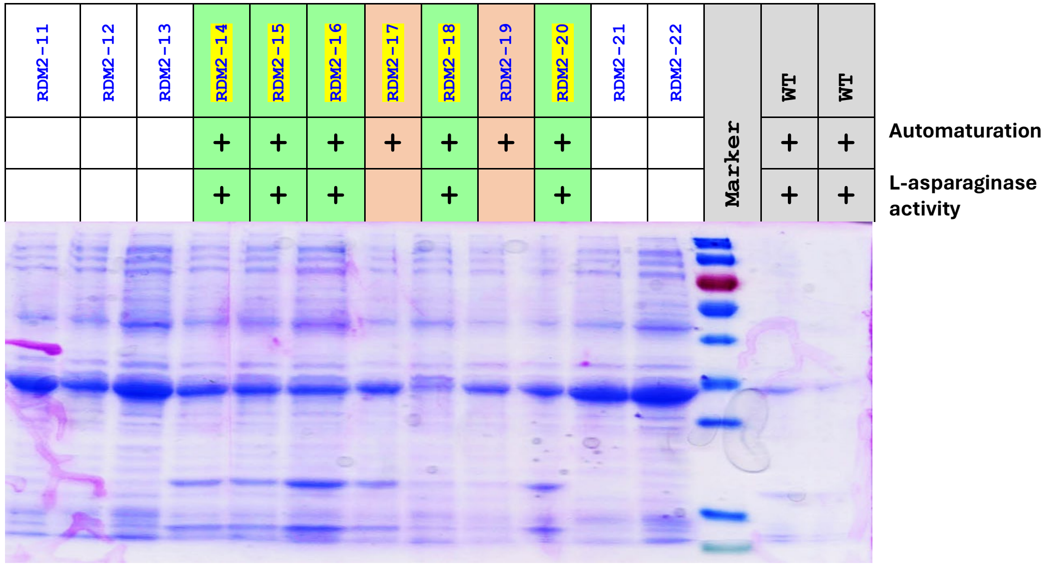

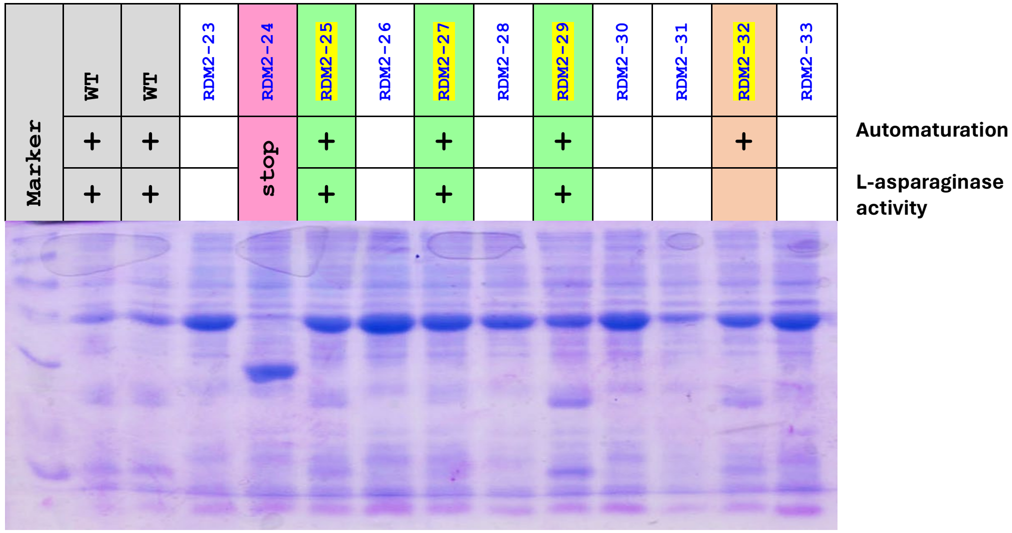

Legend	
Marker	molecular weight SDS-PAGE marker
WT	wild type (WT) EcAIII used as reference
	empty line on the gel
RDMX-Y (stop)	impaired expression caused probably by codon STOP introduced into the sequence
RDMX-Y	variant able to process for subunits α and β but not showing L-asparaginase activity
RDMX-Y	DNA sample of the variant send to sequencing
RDMX-Y	variant cleaved for subunits α and β and showing L-asparaginase activity
RDMX-Y	DNA sample of the variant send to sequencing
RDMX-Y	variant not cleaved for subunits α and β
RDMX-Y	DNA sample of the variant send to sequencing

2. SDS-PAGE gels from screening of RDM3 series (continued on the next page).

	Marker	RDM3-1	RDM3-2	RDM3-3	RDM3-4	RDM3-5	RDM3-6	WT	WT
Automaturation					+			+	+
L-asparaginase activity								+	+

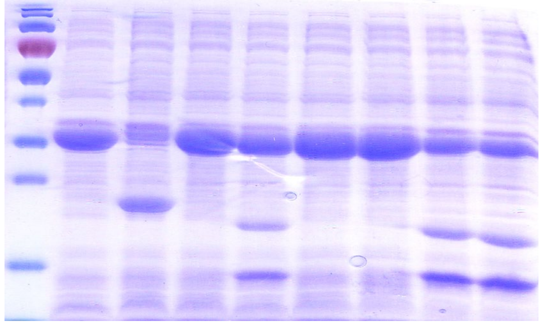

	RDM3-7	RDM3-8	RDM3-9	RDM3-10	RDM3-11	RDM3-12	RDM3-13	RDM3-14	RDM3-15	RDM3-16	Marker	WT	WT
Automaturation						+	stop	stop				+	+
L-asparaginase activity						+						+	+

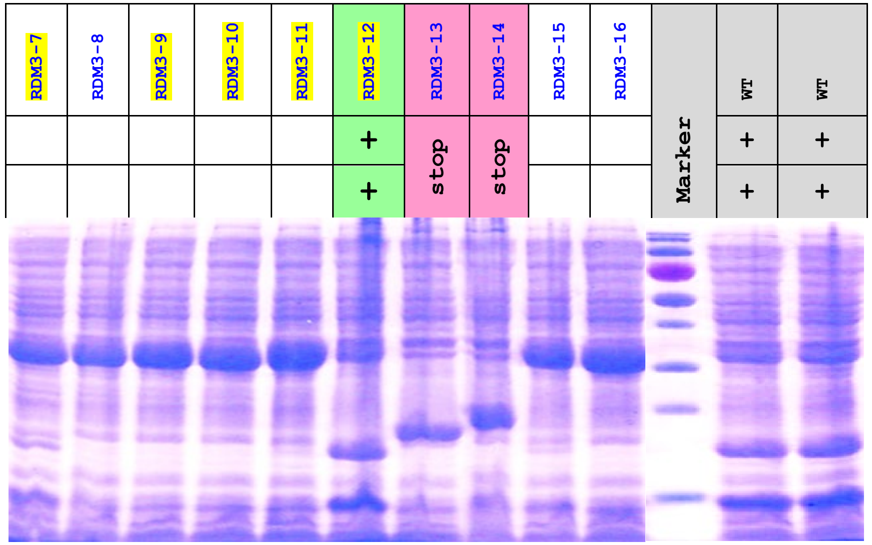

	WT	WT	Marker	RDM3-17	RDM3-18	RDM3-17	RDM3-18	RDM3-19	RDM3-20	RDM3-21	RDM3-22	RDM3-23	RDM3-24	RDM3-25	RDM3-26
Automaturation	+	+				stop		+							
L-asparaginase activity	+	+						+							

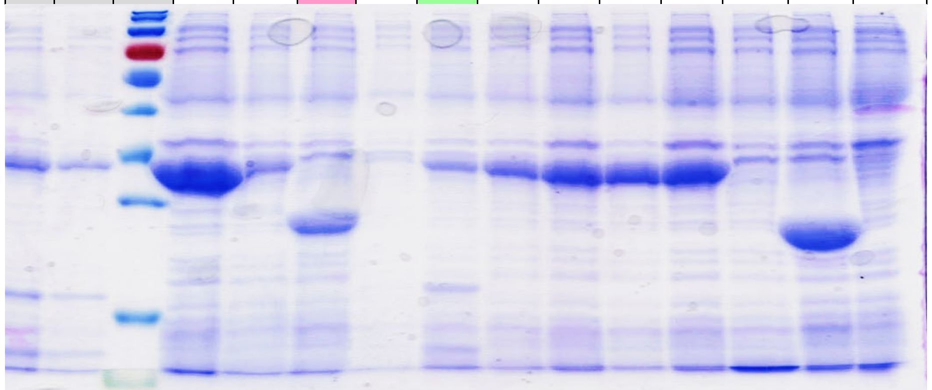

	WT	WT	Marker	RDM3-27	RDM3-28	RDM3-29	RDM3-30	RDM3-31	RDM3-32	RDM3-33	RDM3-34	RDM3-35	RDM3-36	RDM3-37	RDM3-38
Automaturation	+	+													
L-asparaginase activity	+	+													

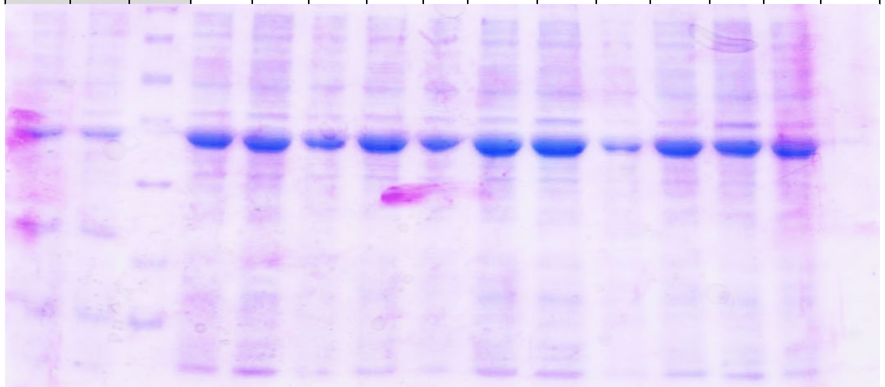

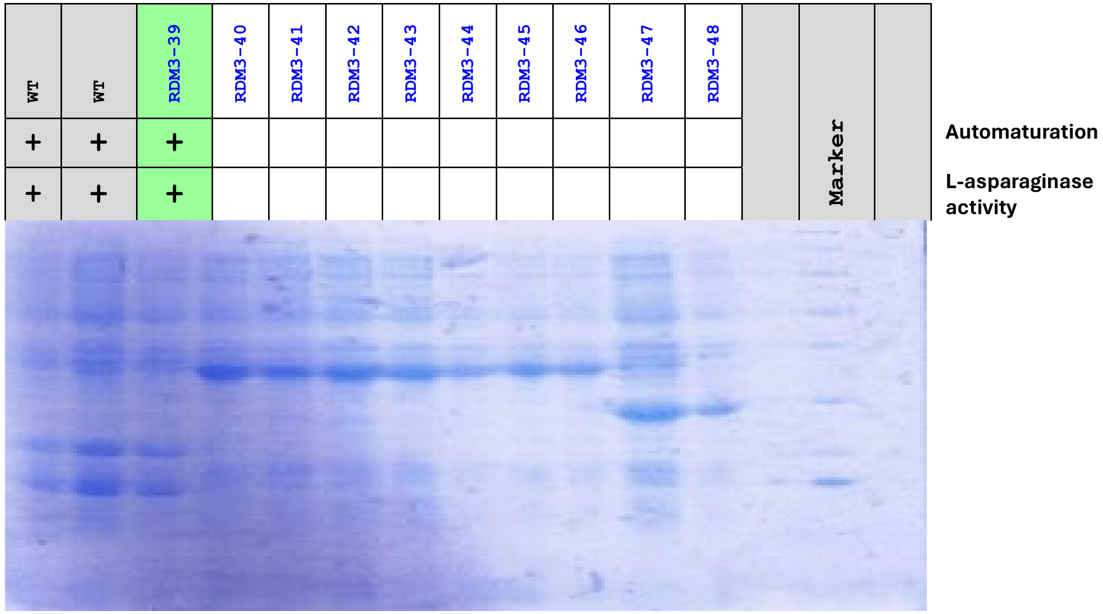
WT
WT
RDM3-39
RDM3-40
RDM3-41
RDM3-42
RDM3-43
RDM3-44
RDM3-45
RDM3-46
RDM3-47
RDM3-48
Marker
Automaturation
L-asparaginase activity
+
+
+
+
+
+

3. SDS-PAGE gels form screening of RDM4 series (continued on the next page).

	Marker	RDM4-1	RDM4-2	RDM4-3	RDM4-4	RDM4-5	RDM4-6	RDM4-7	RDM4-8	RDM4-9	RDM4-10	RDM4-11	WT
Automaturation									stop				+
L-asparaginase activity													+

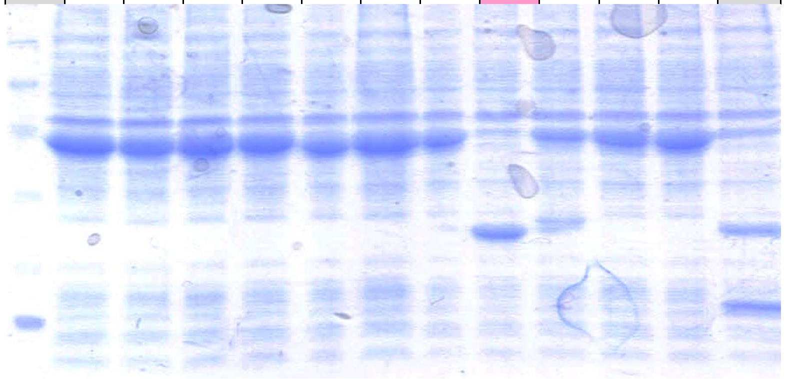

	Marker	WT	RDM4-12	RDM4-13	RDM4-14	RDM4-15	RDM4-16	RDM4-17	RDM4-18	RDM4-19	RDM4-20	RDM4-21	RDM4-22
Automaturation		+			stop								
L-asparaginase activity		+											

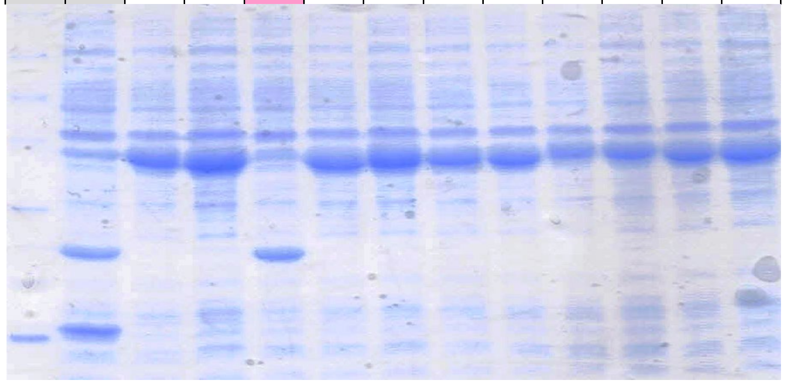

	Marker		RDM4-23	RDM4-24	RDM4-25	RDM4-26	RDM4-27	RDM4-28	RDM4-29	RDM4-30	RDM4-31	RDM4-32	RDM4-33	WT
Automaturation						stop								+
L-asparaginase activity														+

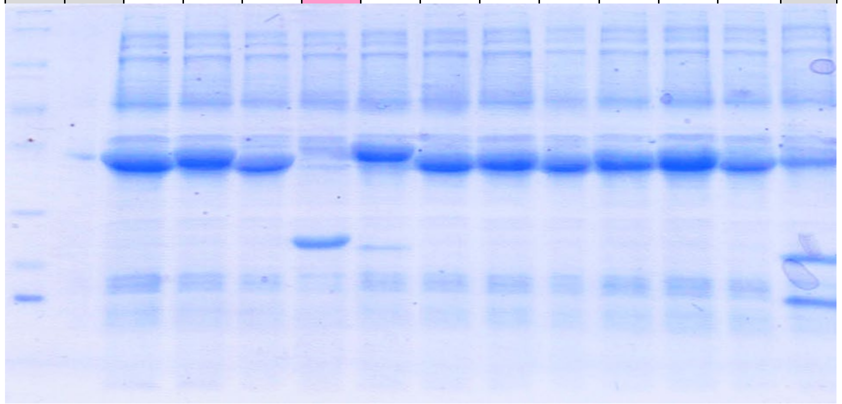

	Marker	WT	RDM4-34	RDM4-35	RDM4-36	RDM4-37	RDM4-38	RDM4-39	RDM4-40	RDM4-41	RDM4-42	RDM4-43	RDM4-44
Automaturation		+			stop		stop						stop
L-asparaginase activity		+											

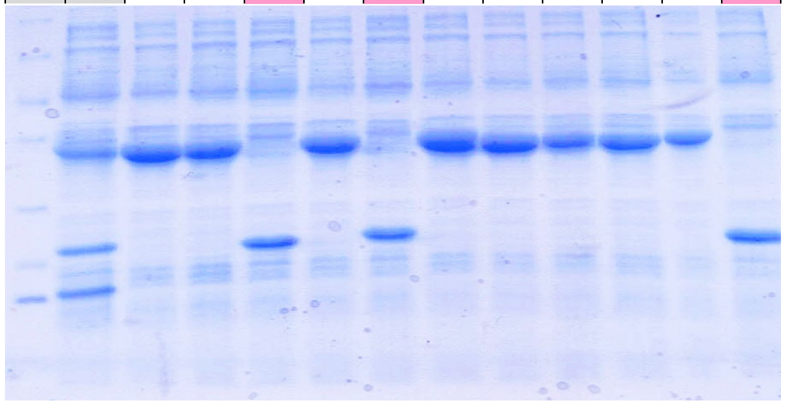

	RDM4-45	RDM4-46	RDM4-47	RDM4-48	RDM4-49	RDM4-50	RDM4-51	RDM4-52	RDM4-53	RDM4-54	RDM4-55	RDM4-56	WT
**Automaturation**	stop			+							+	+	+
**L-asparaginase activity**													+

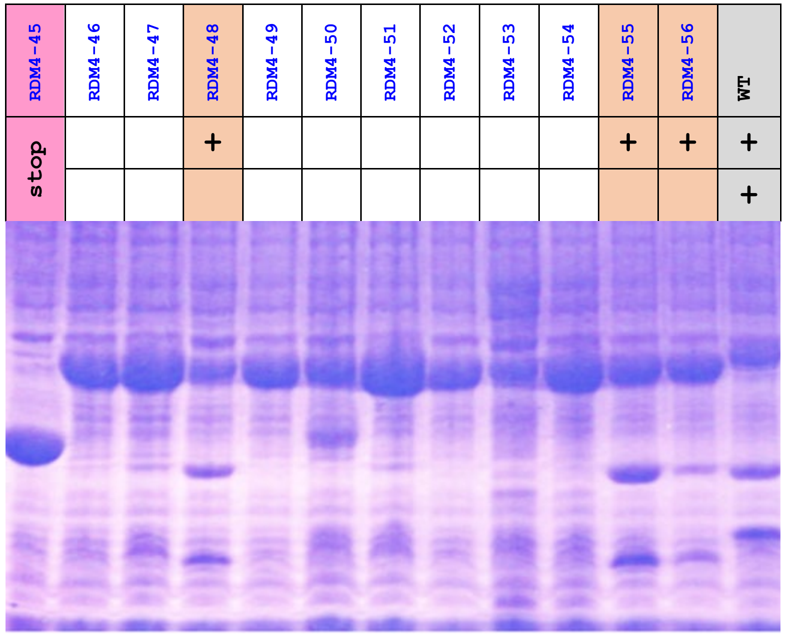

4. SDS-PAGE gels form screening of RDM5 series (continued on the next page).

	RDM5-1	RDM5-2	RDM5-3	RDM5-4	RDM5-5	RDM5-6	RDM5-7	RDM5-8	RDM5-9	RDM5-10	RDM5-11	Marker	WT
Automaturation	+						+						+
L-asparaginase activity	+												+

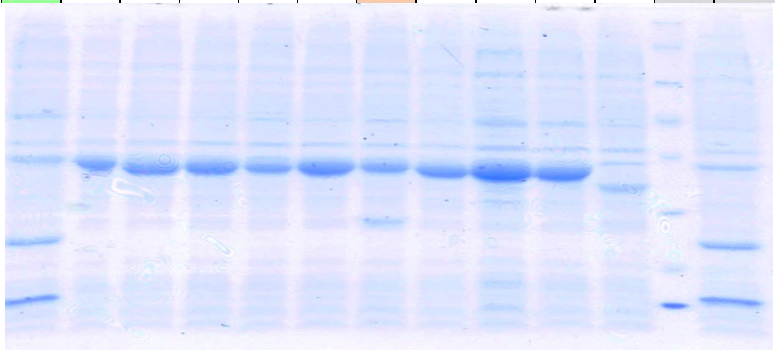

	WT	RDM5-12	RDM5-13	RDM5-14	RDM5-15	RDM5-16	RDM5-17	RDM5-18	RDM5-19	RDM5-20	RDM5-21	RDM5-22	Marker
Automaturation	+		stop					stop					
L-asparaginase activity	+												

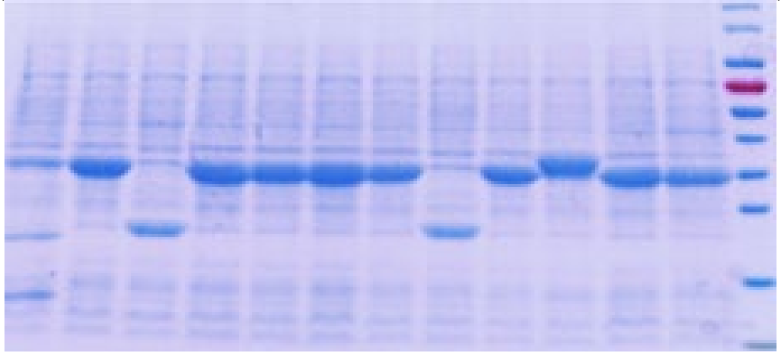

	WT	RDM5-23	RDM5-24	RDM5-25	RDM5-26	RDM5-27	RDM5-28	RDM5-29	RDM5-30	RDM5-31	RDM5-32	RDM5-33	Marker
Automaturation	+						+	+	stop				
L-asparaginase activity	+												

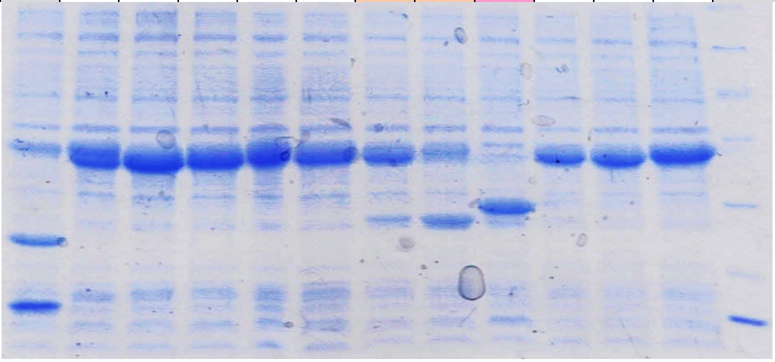

	RDM5-34	RDM5-35	RDM5-36	RDM5-37	RDM5-38	RDM5-39	RDM5-40	RDM5-41	RDM5-42	RDM5-43	RDM5-44	WT	Marker
Automaturation					stop				stop			+	
L-asparaginase activity												+	

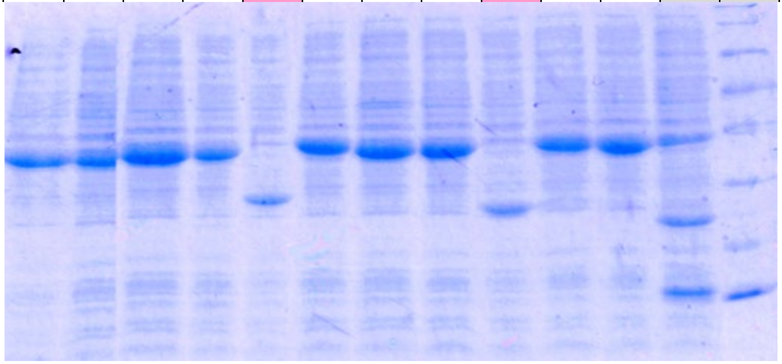

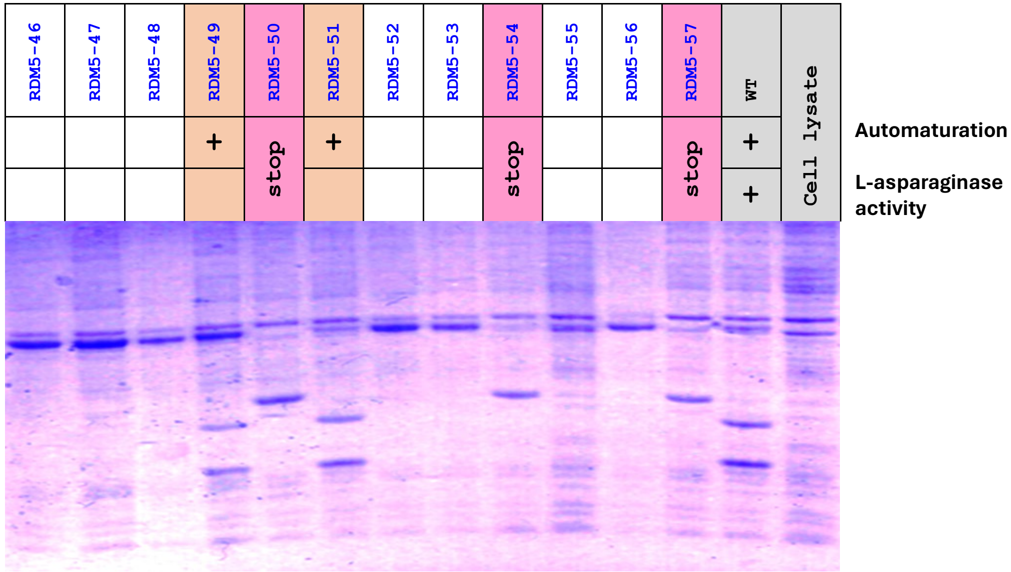
RDM5-46
RDM5-47
RDM5-48
RDM5-49
RDM5-50
RDM5-51
RDM5-52
RDM5-53
RDM5-54
RDM5-55
RDM5-56
RDM5-57
WT
Cell lysate
+
stop
+
stop
stop
+
+
Automaturation
L-asparaginase activity

5. SDS-PAGE gels form screening of RDM6 series (continued on the next page).

	RDM6-1	RDM6-2	RDM6-3	RDM6-4	RDM6-5	RDM6-6	RDM6-7	RDM6-8	RDM6-9	RDM6-10	RDM6-11	RDM6-12	WT	Marker
Automaturation				+									+	
L-asparaginase activity				+									+	

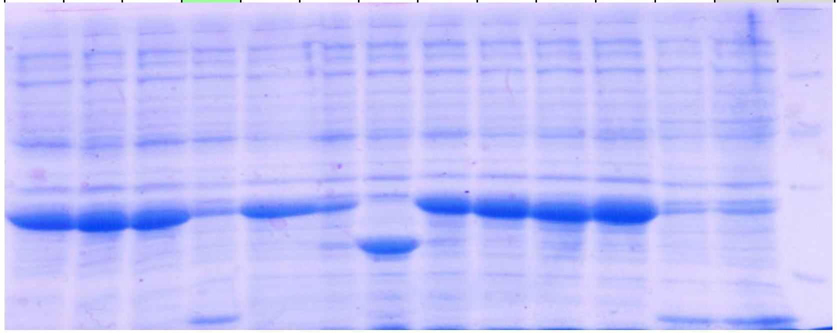

	RDM6-13	RDM6-14	RDM6-15	RDM6-16	RDM6-17	RDM6-18	RDM6-19	RDM6-20	RDM6-21	RDM6-22	RDM6-23	RDM6-24	WT	WT
Automaturation													+	+
L-asparaginase activity													+	+

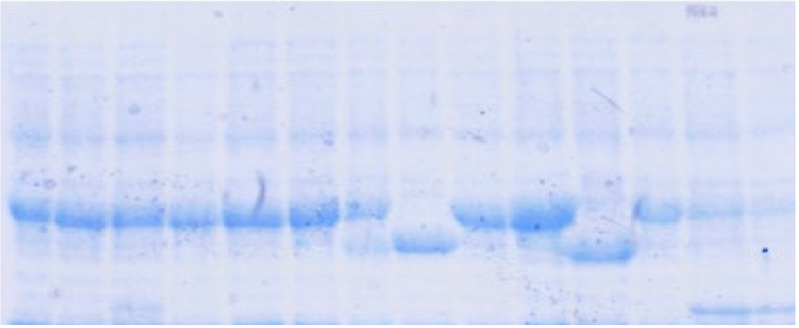

	WT	RDM6-25	RDM6-26	RDM6-27	RDM6-28	RDM6-29	RDM6-30	RDM6-31	RDM6-32	RDM6-33	RDM6-34	RDM6-35	Marker
Automaturation	+				stop							stop	
L-asparaginase activity	+												

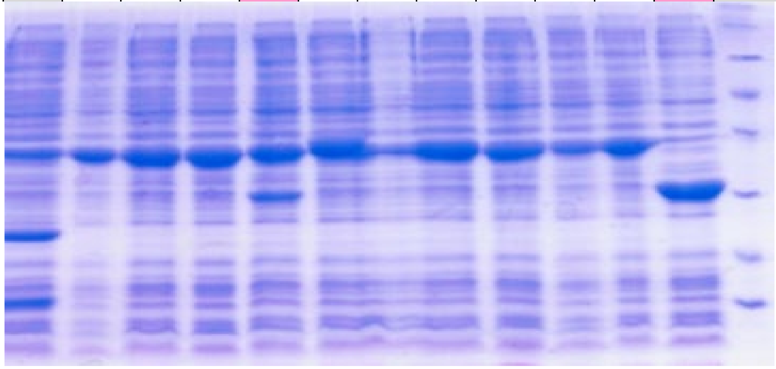

	Marker		WT	RDM6-36	RDM6-37	RDM6-38	RDM6-39	RDM6-40	RDM6-41	RDM6-42	RDM6-43	RDM6-44	RDM6-45	RDM6-46
Automaturation			+									stop		stop
L-asparaginase activity			+											

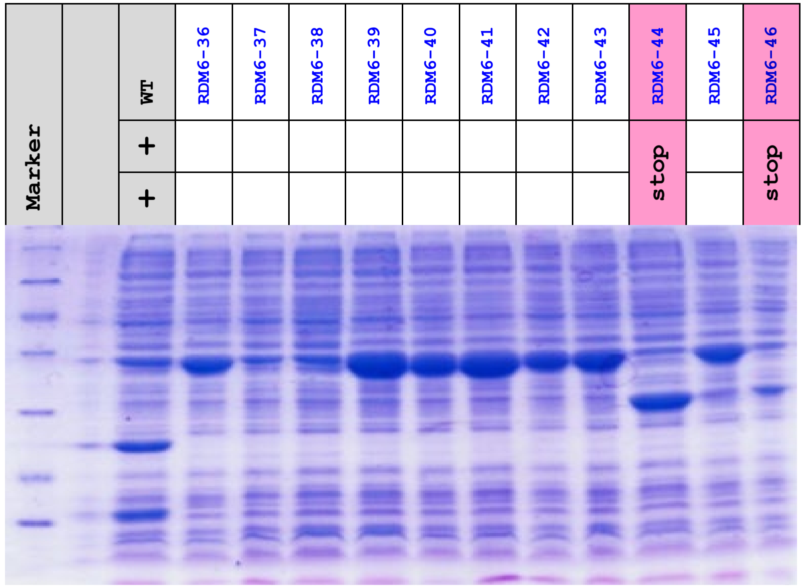

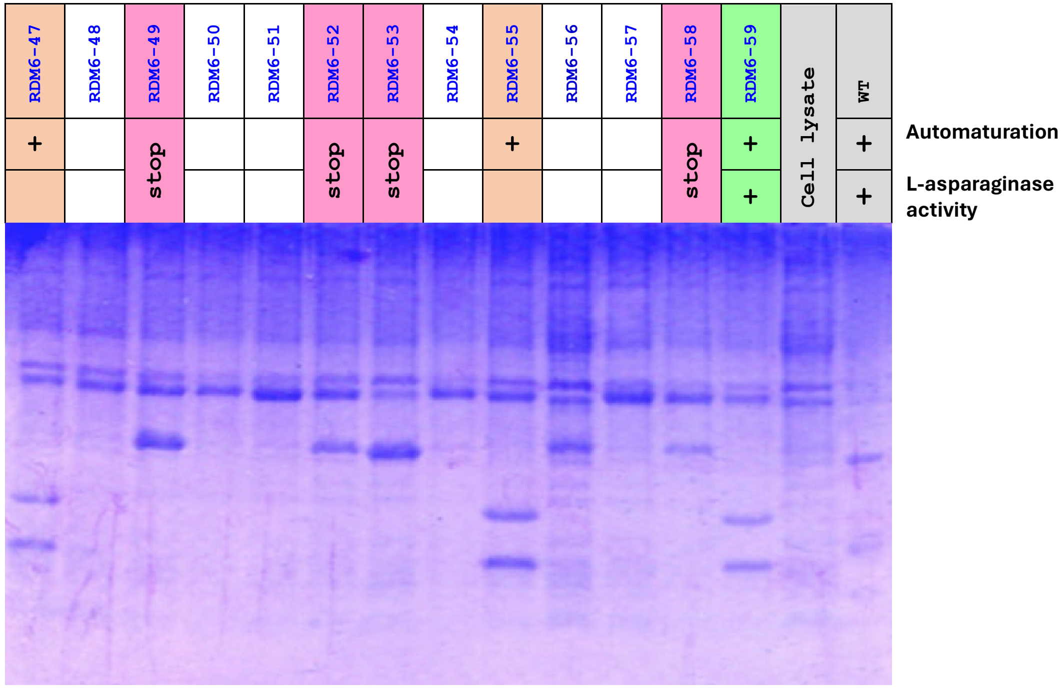
RDM6-47
RDM6-48
RDM6-49
RDM6-50
RDM6-51
RDM6-52
RDM6-53
RDM6-54
RDM6-55
RDM6-56
RDM6-57
RDM6-58
RDM6-59
Cell lysate
WT
Automaturation
L-asparaginase activity
+
stop
+
+
+
+
+

6. SDS-PAGE gels form screening of RDM7 series.

	RDM7-1	RDM7-2	RDM7-3	RDM7-4	RDM7-5	RDM7-6	RDM7-7	RDM7-8	RDM7-9	RDM7-10	RDM7-11	RDM7-12	Marker	WT	Cell lysate
**Automaturation**	+	+	+		+	+	+	+	+	+	+	+		+	
**L-asparaginase activity**	+	+			+	+		+	+	+				+	

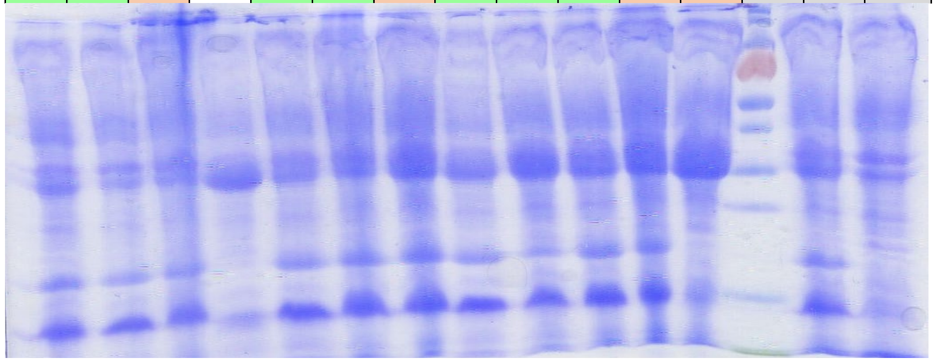

	Marker	RDM7-13	RDM7-14	RDM7-15	RDM7-16	RDM7-17	RDM7-18	RDM7-19	RDM7-20	RDM7-21	RDM7-22	RDM7-23	RDM7-24	RDM7-25	WT	RDM7-26	RDM7-27	Marker	WT
**Automaturation**		+	+	+	+	+	+	+		+			+	+	+	+	+		+
**L-asparaginase activity**			+							+					+	+	+		+

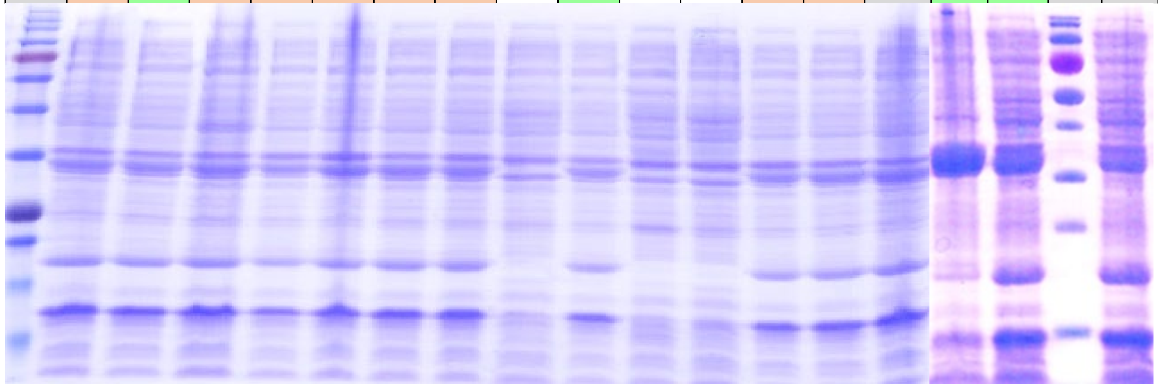

7. SDS-PAGE gels form screening of RDM8 series.

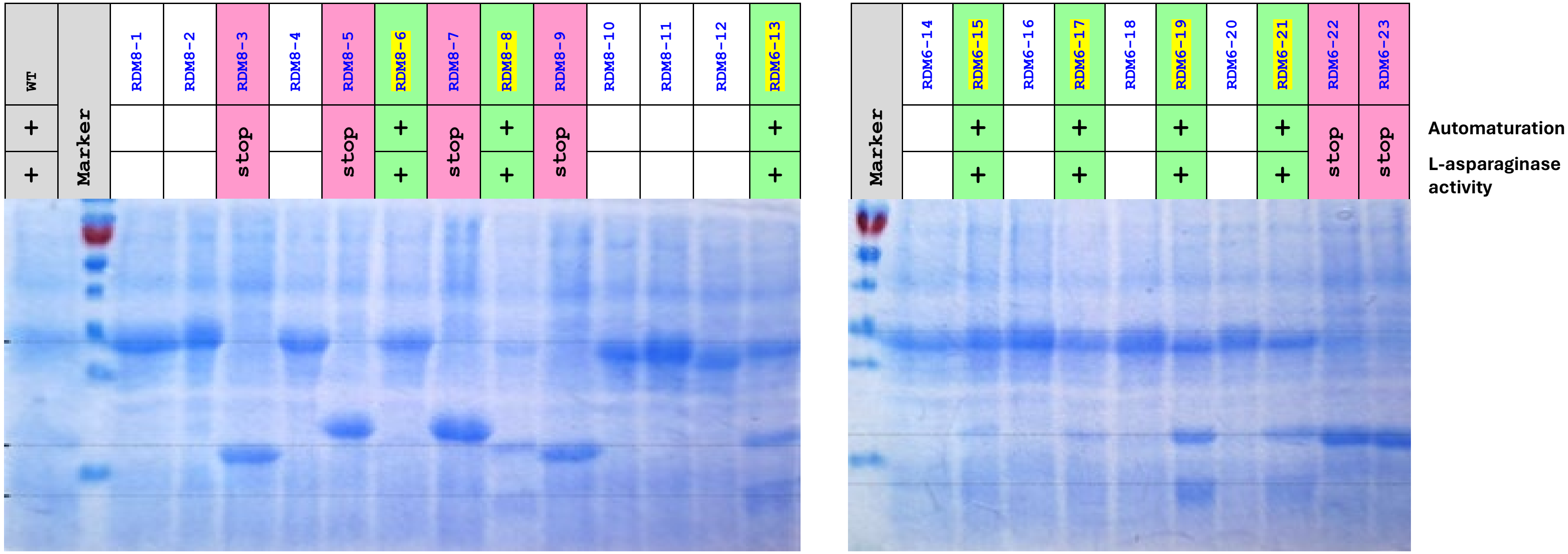

8. SDS-PAGE gels form screening of RDM9 series.

	RDM9-1	RDM9-2	RDM9-3	RDM9-4	RDM9-5	RDM9-6	RDM9-7	RDM9-8	RDM9-9	RDM9-10	RDM9-11	RDM9-12	WT	Inactive mutant T179A	Marker
**Automaturation**							+						+		
**L-asparaginase activity**													+		

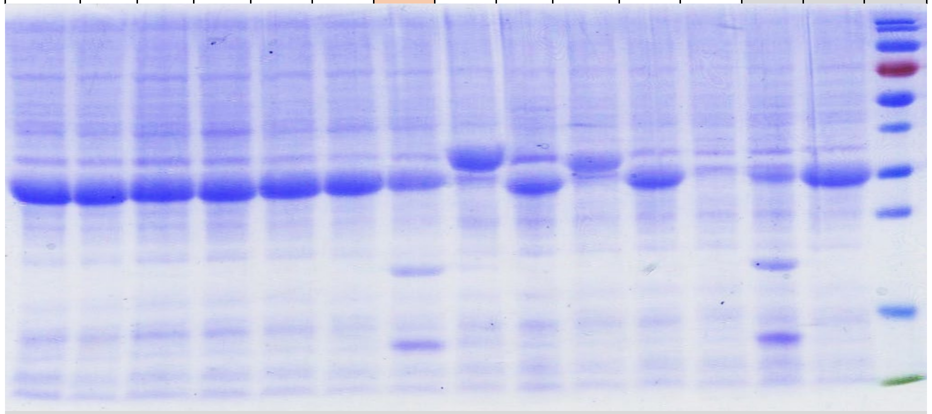

	WT	WT	RDM9-13	RDM9-14	RDM9-15	RDM9-16	RDM9-17	RDM9-18	RDM9-19	RDM9-20	RDM9-21	RDM9-22	RDM9-23	RDM9-24	Marker
**Automaturation**	+	+							+						
**L-asparaginase activity**	+	+							+						

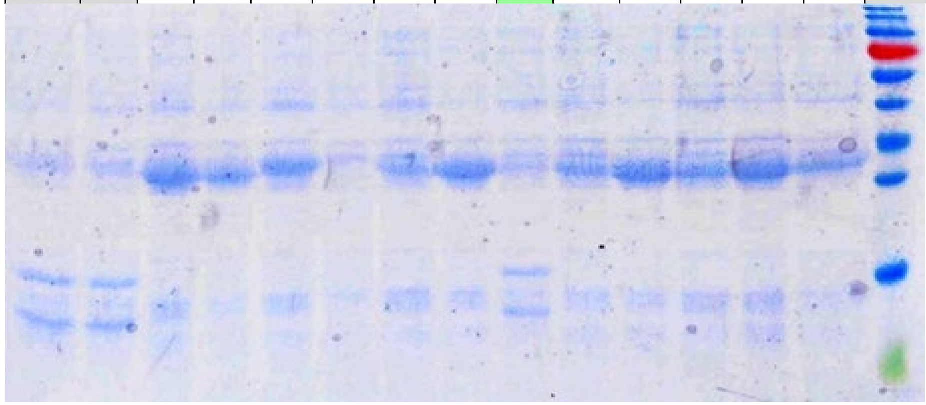

Supplement: Supplementary file 1 [file DataSheet1.pdf]
